# Online Food Frequency Questionnaire From the Cohort of Universities of Minas Gerais (CUME Project, Brazil): Construction, Validity, and Reproducibility

**DOI:** 10.3389/fnut.2021.709915

**Published:** 2021-09-23

**Authors:** Heloísa Gambarelli de Araújo Azarias, José Luiz Marques-Rocha, Aline Elizabeth da Silva Miranda, Luana Caroline dos Santos, Ana Luíza Gomes Domingos, Helen Hermana Miranda Hermsdorff, Josefina Bressan, Fernando Luiz Pereira de Oliveira, Arieta Carla Gualandi Leal, Adriano Marçal Pimenta

**Affiliations:** ^1^Postgraduate Program in Nutrition and Health, School of Nursing, Universidade Federal de Minas Gerais (UFMG), Belo Horizonte, Brazil; ^2^Department of Integrated Education of Health, Universidade Federal do Espírito Santo (UFES), Vitória, Brazil; ^3^Department of Nutrition, School of Nursing, Universidade Federal de Minas Gerais (UFMG), Belo Horizonte, Brazil; ^4^Postgraduate Program in Nutritional Science, Department of Nutrition and Health, Universidade Federal de Viçosa (UFV), Viçosa, Brazil; ^5^Department of Nutrition and Health, Universidade Federal de Viçosa (UFV), Viçosa, Brazil; ^6^Department of Statistics, Universidade Federal de Ouro Preto (UFOP), Ouro Preto, Brazil; ^7^Department of Maternal and Child Nursing and Public Health, School of Nursing, Universidade Federal de Minas Gerais (UFMG), Belo Horizonte, Brazil

**Keywords:** reproducibility of results, validation studies, surveys and questionnaires, food frequency questionnaire, internet

## Abstract

**Background:** The Food Frequency Questionnaire (FFQ) is usually used in epidemiological studies to assess food consumption. However, the FFQ must have good accuracy, requiring its validation and reproducibility for the target population. Thus, this study aimed to describe the construction of the online Food Frequency Questionnaire (oFFQ) used at the Cohort of Universities of Minas Gerais (CUME project, Brazil) and evaluate its validity and reproducibility.

**Methods:** The oFFQ was answered two times in 1 year (March/August 2018—March/April 2019; *n* = 108 participants—reproducibility), and four 24-h dietary recalls (24hRs) were applied in two seasons of the southern hemisphere [two 24hRs in autumn (March/June 2018) and two 24hRs in winter (August/September 2018); *n* = 146 participants—validity]. To assess the validity and reproducibility, the intraclass correlation coefficients (ICCs) were estimated.

**Results:** The oFFQ had 144 food items separated into eight groups (dairy products; meat and fish; cereals and legumes; fruits; vegetables; fats and oils; drinks; other foods). In assessing the validity, ICCs for energy and macronutrients were considered moderate, ranging from 0.41 (energy) to 0.59 (protein), while the ICCs for micronutrients were considered low to moderate, ranging from 0.25 (fibers) to 0.65 (vitamin B6). Regarding reproducibility assessment, ICCs for energy and all the assessed items were considered moderate to excellent, ranging from 0.60 (vegetables) to 0.91 (vitamin E and retinol).

**Conclusions:** The self-reported oFFQ had satisfactory validity and reproducibility. So, it can be used to analyze the association between food consumption and chronic diseases in the participants of the Cohort of Universities of Minas Gerais (CUME project—Brazil).

## Introduction

Food consumption evaluation has been commonly used in epidemiological studies since it correlates with health and chronic disease determinants (1, 2). However, there are countless challenges related to this process, including intra- and interpersonal variabilities, interviewer and/or interviewee bias, and adherence to assessment instruments.

In recent years, face-to-face data collection has decreased in epidemiological studies worldwide (3), parallel with an increase in Internet access (4). Thus, the development of instruments in the virtual environment for data collection has become a promising trend (4), including assessing food consumption (5), mainly when large populations are involved.

Online data collection allows sending alerts to users, reducing the study load for participants, and maintaining a distance between researchers and subjects, limiting self-censorship in the interview. In addition, it facilitates processing and causes greater reliability of information due to the multimedia support and elimination of steps related to data entry or scanning of paper forms (6, 7).

However, there are still few epidemiological studies in Brazil that have used online questionnaires for food consumption assessment (8, 9). In this context, the present study, the Cohort of Universities of Minas Gerais (CUME project, Brazil), aimed to evaluate the relationship between the Brazilian food pattern and the nutritional transition on non-communicable diseases (NCDs) in higher education graduates of federal universities located in the state of Minas Gerais, Brazil (10).

To achieve the objective of the CUME project, an online and self-administered Food Frequency Questionnaire (oFFQ) has been used. This method demonstrates the practicality in obtaining and analyzing data, at a low cost, and the possibility of investigating food consumption over a long period (2, 11, 12).

On the other hand, the Food Frequency Questionnaire (FFQ) has limitations that include the use of a previously defined list of foods, dependence on the memory of previous eating habits, and difficulty in establishing the precise amount of food consumed (13). Besides, the FFQ must have good accuracy, requiring its validation for the target population; the use of data collection instruments previously validated in other studies is not recommended. In addition, the FFQ must have good reproducibility to increase the guarantee that the data provided by the interviewees are not due to chance.

Thus, we aimed to describe the construction of the oFFQ used at the CUME project and evaluate its validity and reproducibility.

## Materials and Methods

### CUME Project

The CUME project is an open population cohort, which has been developed with graduates from institutions of higher education in the State of Minas Gerais (Brazil), whose design, dissemination strategies, and profile of the first baseline participants were previously reported (10).

The choice of target population for the CUME study was because participants with a high level of education, in general, report data that are considered as more reliable (14).

The baseline data collection of the CUME project was carried out between March and August, 2016 (first wave), and March and August, 2018 (second wave) with alumni from Universidade Federal de Viçosa (UFV), Universidade Federal de Ouro Preto (UFOP), Universidade Federal de Lavras (UFLA), and Universidade Federal de Juiz de Fora (UFJF), and Universidade Federal de Minas Gerais (UFMG), graduated between 1994 and 2017.

The participants of the CUME project had access to the baseline questionnaire divided into two parts separately with a 1-week interval. The first part consisted of questions related to lifestyle, sociodemographic, anthropometric, biochemical, and clinical data; individual and family mentioned morbidity: use of medication, and personal examination history. In the second part, participants completed the oFFQ.

### Construction of the Online Food Frequency Questionnaire

The construction of oFFQ was based on the original version of the quantitative instrument previously validated for the Brazilian population, containing a list of 135 food items to assess the association of food consumption in the last year with NCDs in epidemiological studies (15).

Then, we included food items that are significantly consumed by our target population and representatives of all the regions of Brazil (16). These food items were prato and canastra Brazilian cheeses, cottage cheese, lard, cod, chard, mate/black teas, white/green teas, commercially processed juice (light and diet), and light sugar. In addition, the nomenclature of some foods was adapted to suit better the language used in different regions of the country. Food items that indicated food brands were modified to generic names. Foods that were not common in our target population diet, such as radiche, morcilla, and keschmier, were excluded. Finally, food items such as “canned fruit juices/tetra brik/with sugar” and “sweetened artificial juices” were merged to the item “processed fruit juice (canned/box/powder),” while items such as “black coffee,” “espresso,” “cappuccino,” and “soluble coffee” were merged to the item “coffee.”

Food portions of the oFFQ were expressed in homemade measures commonly used by Brazilians (teaspoon, tablespoon, ladle, pinch, tong, saucer, cup, and glass) or in traditional food portions (unit, slices, and pieces) (17). Each food item had one to three serving options. In addition to the information about the food portion, the questionnaire had sections regarding the frequency of food consumption (from one to nine or more) as units of time (day, week, month, or year).

At the end of the oFFQ, more questions were added to learn about the eating habits and practices of the participants that may influence the risk or protection related to NCDs, such as number of meals per day; visible fat meat intake; addition of salt and/or sugar to ready meals; consumption of organic foods, lactose-free foods, gluten-free foods, probiotics, and prebiotics; and use of dietary supplements. In addition, explanatory notes for technical terms have been included to facilitate a better understanding when necessary.

#### Face and Content Validation of the Baseline Questionnaire and Pilot Studies

A validation of face and content was carried out to evaluate the baseline questionnaire of the CUME concerning comprehensiveness and complexity of understanding, relevance, applicability, clarity, success possibility, absence of bias, items not included, and extension. For this stage, five nutrition researchers from the UFV, UFMG, and the UFOP were invited to evaluate the instrument.

Moreover, two pilot studies were carried out to evaluate the data collection instrument. First, the printed version of the self-completed questionnaire was tested with 25 alumni from the UFV and the UFMG from different training areas. Then, the auto-filling online version of the instrument was developed in the virtual environment for data collection of the CUME project; it was also evaluated by other 26 former students from the UFV and the UFMG.

At the end of the questionnaire, participants had an open space to write some observations and suggestions that the researchers appreciated. We divided the data collection into two parts, leaving the oFFQ in the second part to facilitate its completion and increase the participant compliance. In addition, a photographic album of food portions and utensils was prepared to help estimate the portion size and complete the questionnaire.

#### Online Photo Album of Food Portions and Utensils

The photographic record was carried out in August 2015, at the Laboratory of Energy Metabolism and Body Composition (LAMECC) of the Department of Nutrition and Health at the UFV.

This photo album was based on food portions and utensils that were used in the oFFQ. Weights (in grams) for small, medium, and large portions were defined according to the Brazilian tables containing weights for food portions and home measures (17, 18). However, due to the lack of tables for some foods, such as meats, fruits, and vegetables, these were adapted and weighted at LAMECC using a portable precision scale (BS 3000A, Bioprecisa, Curitiba, Brazil) with a 3,000 g capacity and 0.1 g sensitivity. In addition, small and medium portions were considered 50 and 75%, respectively, of the weight of the large portion (19), with a variation of up to 30%.

To elaborate the online photo album, foods were pre-prepared (cleaned, peeled, and cut) and prepared according to good food handling practices to guarantee the quality of the food and photographs. The prepared foods were divided into portions, similar to those presented in the oFFQ. All prepared foods were placed on white porcelain, with aluminum cutlery or in glasses, and immediately photographed to avoid the loss of sensory characteristics.

The photographs were taken with a standardized neutral color background, using a three-dimensional digital camera (Cyber-Shot DC-WX 100, 18.2 megapixels, Sony Brand, Manaus, Brazil). In addition, all photos were marked with a watermark using the logo of the CUME project.

A total of 42 food items and utensils were photographed individually at different angles and from different distances. In addition, 800 photographic images of food items and 160 utensils were obtained. We carefully assessed and standardized the angle and distance, selecting the photographs that allowed better detail of the portion size and the utensil.

Thus, the photo album consisted of 96 food photographs, with 9 pictures of the dairy group (yogurt, curd, and cheese), 16 of the cereal group (polenta, lasagna, pizza, macaroni, and cheese bread), 15 of meat and fish (ham, beefsteak, diced beef, chunk chicken, salmon, and fish slices), 20 of fruits (pineapple, banana, peach, avocado, guava, orange, apple, papaya, mango, grape, watermelon, and melon), 16 of vegetables (potatoes, cucumbers, tomatoes, and lettuce), two of beverages (juice and wine), and 18 of the group called “other foods” [popcorn, peanuts, chocolate, ice cream, pie, coxinha (Brazilian deep-fried dumpling filled with shredded chicken), pudding]. Five photographs of the utensils [cup, glass (aperitif), tablespoon, soup spoon, and ladle] complemented the material.

For those food items that were not included in the online photo album, a photo of another food item with a similar portion size or of the same nature was presented [e.g., one participant consumed a glass of whole milk, they would use the photograph of the juice portions (including the glass) as a reference]. The photos were organized to provide better visibility and comparability.

A complete Portuguese version of the oFFQ could be accessed in http://www.projetocume.com.br/questionario [Questionário da linha de base (Q_0).pdf—pages 37–162].

### Online Food Frequency Questionnaire Validation and Reproducibility

#### Sample and Data collection

A total of 1,357 graduates answered the baseline questionnaire between March and August 2018. At the end of each week, the virtual platform developed for the CUME project automatically provided a report with the names and e-mail addresses of the participants who had completed the data collection. Subsequently, we randomly selected 150 participants and sent an invitation by email to study the validity and reproducibility of the oFFQ, informing the objectives and procedures to be used.

To the validity of the oFFQ, the 24-h dietary recall (24hRs) was used as a reference method for comparison. The participants who responded positively to the study invitation were contacted by cell phone in two different seasons of the year in the southern hemisphere to guarantee the variability of food consumption that occurs throughout the year due to climate change. In the first moment, two 24hRs were carried out on two random and alternate days of the week (from Monday to Friday) between March 20 and June 21 (autumn), 2018. In the second moment, the other two 24hRs were also carried out on two random and alternate days of the week (from Monday to Friday) between August 20 and September 23 (winter), 2018.

In both periods, the 24hRs were conducted by previously trained interviewers following the Multiple-Pass method (20). Participants were asked to report all food consumption from the day before the call, describing each meal, time, place, and then detailing food quantities. At the end of the 24hRs, a review of the information and investigation of possible unreported items was carried out. We also attached our online photo album of the oFFQ food items and utensils to the study invitation e-mail, the same one used in the virtual data collection platform of the baseline questionnaire, named in this study as oFFQ1. The interviews were written down on paper.

In the first moment, the 24hRs were applied to 150 participants. From these, four participants were excluded because they reported inconsistent energy consumption (<500 kcal/day or >6,000 kcal/day) (21), resulting in a sample of 146 participants. In the second moment, 12 participants did not respond to the interviewers after five telephone contact attempts. Thus, the 24hRs were performed only with 134 participants.

**Figure 1:**
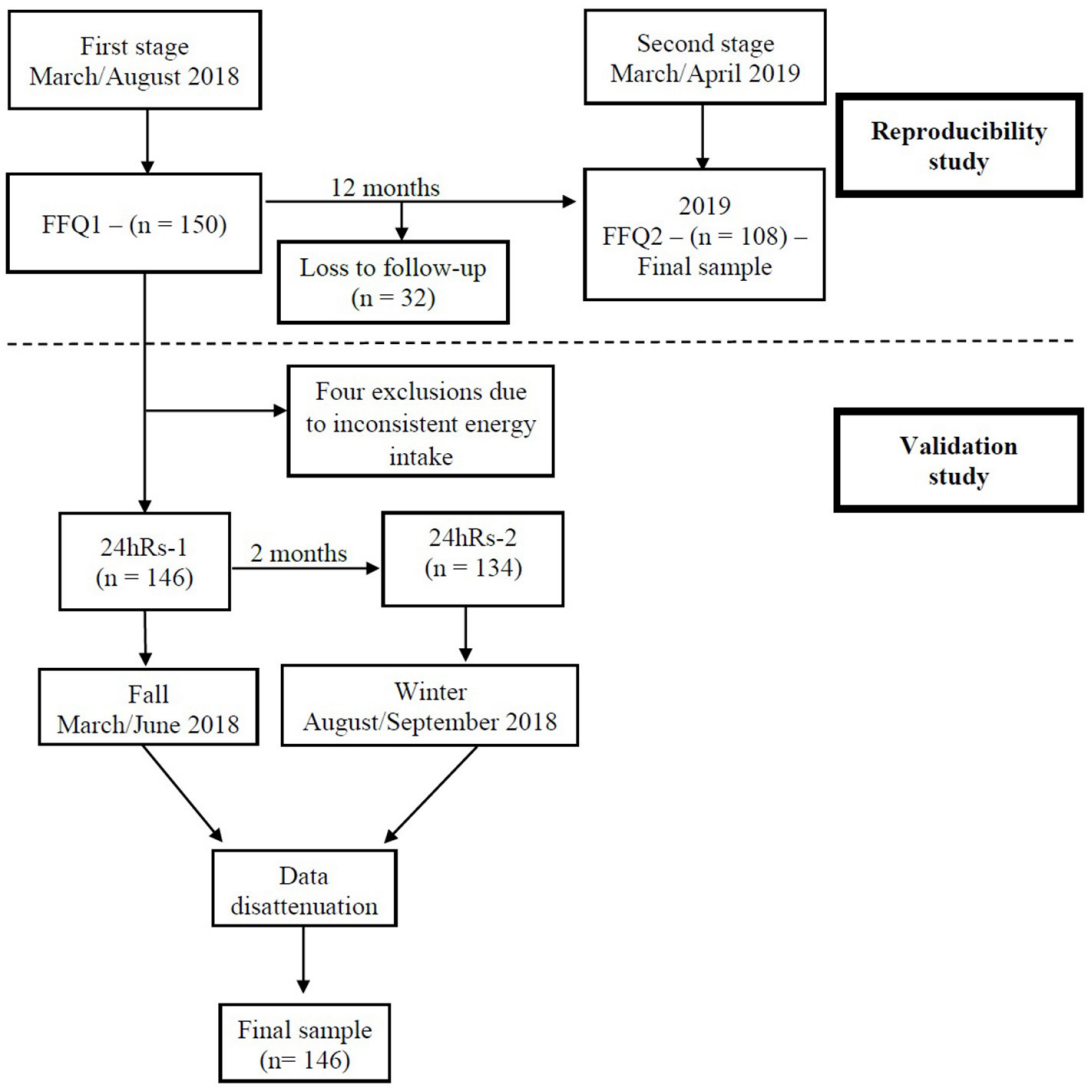
Study flowchart of the validity and reproducibility of the online food frequency questionnaire. CUME Project, 2019.

For reproducibility, participants in the validity stage received an access link to the oFFQ on the virtual platform of the CUME project to fill the instrument again. As a result, between March and April 2019, 108 participants answered the oFFQ in a completely new way, referred to in this study as oFFQ2 (Figure 1).

#### Food Consumption Analysis

Intake of each food item from the oFFQ or the four 24hRs was transformed into daily consumption (grams or milliliters), multiplying the number of portions (one to nine) by the portion size (in grams), and then dividing it by the consumption frequency (daily: 1; weekly: 7; monthly: 30; or yearly: 365).

Caloric intake, nutrients (carbohydrates, proteins, fats, vitamins, and minerals), and other specific components (fibers, carotenoids, and sugars) were calculated according to the nutritional composition of each source food provided by the Household Budget Survey (22), with the help of Excel (version 2010) and SPSS (version 19). Furthermore, the food items were separated in two ways: (a) according to the eight food groups present in the oFFQ, which were organized according to nutritional similarity (dairy products; meat and fish; cereals and legumes; fruits; vegetables; fats and oils; beverages; other foods) and (b) according to the NOVA classification (23), which divides foods according to the degree of industrial processing into four groups: in natura/minimally processed, culinary ingredients, processed, and ultra-processed (Supplementary Table 1).

### Data Analysis

The sample was characterized with the distribution of frequencies or means (SDs) of the sociodemographic (age, skin color, marital status, education level, regular work in the last 12 months), anthropometric (BMI = weight/height^2^), and lifestyle variables [smoking (no, ex-smoker, smoker) and alcohol consumption (no, yes)].

The values of energy consumption, nutrients, food groups according to nutritional similarity and food groups according to the degree of industrial processing derived from the 24hRs were disattenuated (corrected by intra-individual variability), generating unique values for each item to participants who responded four (*n* = 134) or only two (*n* = 12) 24hRs. We used the PC-SIDE program (Department of Statistics, Iowa State University, Iowa, United States), developed by the National Research Council and Iowa State University (24, 25). Thus, each participant had a unique value, possibly using the whole sample (*n* = 146) to this data analysis of validation study. Moreover, consumption values were adjusted for energy intake by the residual method (26).

The means and SDs of the values of energy consumption, nutrients, food groups according to nutritional similarity, and food groups according to industrial processing degree were calculated for the estimates derived from the oFFQ1, 24hRs, and oFFQ2.

For the oFFQ validity and reproducibility analyses, ICCs were calculated between the consumption values derived from the oFFQ1 and 24hRs, and from the oFFQ1 and oFFQ2, respectively. The ICCs were classified as excellent (≥0.75), moderate (≥0.40 to <0.75), and low (<0.40) (27).

The consumption of energy, nutrients, food groups according to nutritional similarity, and food groups according to industrial processing degree of all the participants estimated by the oFFQ1, 24hRs, and oFFQ2 were categorized into tertiles, evaluating the percentage of agreement between the measurements, being considered: exact (when the participants consumed the item evaluated in the same tertile when compared between oFFQ1 and 24hRs or between oFFQ1 and oFFQ2); adjacent (adjacent tertiles) and discordant (opposite tertiles).

Finally, to assess the differences between the values of energy, nutrients, and food group consumption between the oFFQ1 and the 24hRs, the method proposed by Bland and Altman (28) was used. For this, we constructed scatter plot graphs with absolute differences between the values of oFFQ1 and 24hRs (oFFQ1 – 24hRs) on the *y*-axis and the mean of the values obtained by oFFQ1 and 24hRs [(oFFQ1 + 24hRs)/2] on the *x*-axis.

Moreover, we chose three nutrients with higher (vitamin B6, calcium, and vitamin D) and lower (fibers, added sugar, and sodium) ICC values compared with oFF1 and 24hRs to display the results of the Bland and Altman method of concordance analysis. All statistical analyses were conducted in the SPSS program (version 19) at a significance level of 5%.

## Results

Approximately 146 participants (66.4% women; 34.4 ± 8.6 years old) from CUME were included in this study (Table 1). Most of them declared to be white, with individual incomes of up to five minimum wages and full-time jobs. Regarding lifestyle, 8.2% reported smoking, 70.5% consumed alcoholic beverages, and 78.8% practiced physical activity at least once a week. In addition, overweight (BMI ≥ 25.0 kg/m^2^) was observed in 40.4% of the participants.

**Table 1:** General characteristics of participants selected for validity and reproducibility of the food frequency questionnaire (*n* = 146 participants).

**Characteristics**	**Total** **(***n*** = 146)**	
Gender^*^		
Female	97	66.4
Male	49	33.6
Age (years)^**^	34.4 ±8.6	
Skin color^*^		
White	103	70.5
Black/brown	43	29.5
Marital status^*^		
Single	72	49.3
Married/stable union/others	72	49.3
Separated/divorced	2	1.4
Education level^*^		
Bachelor	43	29.5
Additional specialization	31	21.2
Master's	51	34.9
PhD/post-doctoral	21	14.4
Study field^*^		
Health/biological sciences	42	28.8
Engineering	24	16.4
Quantitative sciences	16	10.9
Agricultural sciences	23	15.8
Social sciences/linguistics/Art	41	28.1
Professional status^*^		
Full time/part-time/informal	106	72.6
Student	28	19.2
Unemployed	12	8.2
**Individual income (minimum wage)** ^*^ ^†^		
<5 times the minimum wage	69	57.0
≥5 to <10 times the minimum wage	35	28.9
≥10 times the minimum wage	17	14.1
**Smoking habit**		
No	116	79.5
Ex-smoker	18	12.3
Yes	12	8.2
Alcohol consumption^*^		
No	43	29.5
Yes	103	70.5
Physical activity^*^		
No	31	21.2
Yes	115	78.8
BMI (kg/m^2^)^*^	24.8	4.3
Normal weight	87	59.6
Overweight	59	40.4

*CUME Project, 2019*.

* *Data are the mean ± standard deviation*,

** *absolute frequency (percentage)*;

† *minimum wage (R﹩ 954.00 in 2018)*.

Regarding the validity of the oFFQ, most of the means of energy, nutrients, and food group consumption in the oFFQ1 were higher than those measured in the 24hRs. Overall, an agreement between the oFFQ1 and the 24hRs was moderate, with an average ICC of 0.44 and the exact + adjacent percentage agreement of 88.1%. Energy and all macronutrients also showed moderate agreement between the instruments, with the following ICCs: 0.41 for energy, 0.50 for carbohydrates, 0.51 for lipids, and 0.59 for proteins. Variations between micronutrients for ICC values were 0.28 (sodium) to 0.65 (vitamin B6). According to nutritional similarity, the ICCs ranged from 0.34 (fats and oils) to 0.62 (fruits) for food groups. Concerning the degree of industrial processing, the concordances were moderate for the ultra-processed foods (ICC = 0.60) and processed (ICC = 0.54) groups, and low for the group of in natura/minimally processed foods (ICC = 0.36) and the group of culinary ingredients (ICC = 0.36) (Table 2).

**Table 2:** Mean and SD of daily consumption, intraclass correlation coefficient (ICC), and percentage (%) of concordance between the online food frequency questionnaire (oFFQ) and the 24-h dietary recalls (24hRs) (*n* = 146 participants).

**Energy and nutrients** **(***n*** = 146)**	**Averages (SD)**		**ICC**	**Concordance (%)**		
	oFFQ1^*^	24hRs^**^	**Disattenuated and adjusted**	**Exact**	**Exact + adjacent**	**Discordant**
Energy (kcal)	2293.54 (785.16)	2202.16 (323.77)	0.41	43.7	85.2	14.8
Lipids (g)	94.48 (17.44)	89.98 (8.14)	0.51	52.1	92.3	7.7
Proteins (g)	103.02 (27.38)	94.16 (11.91)	0.59	44.4	90.9	9.1
Carbohydrates (g)	258.43 (54.53)	246.06 (26.70)	0.50	49.3	83.1	16.7
Fibers (g)	28.40 (8.67)	20.91 (4.15)	0.25	48.5	91.2	9.2
Calcium (mg)	862.01 (290.35)	749.47 (184.97)	0.61	46.5	89.5	10.5
Phosphorus (mg)	1473.05 (303.98)	1296.99 (187.56)	0.47	43.4	94.4	5.6
Iron (mg)	12.149 (1.99)	10.99 (0.96)	0.32	43.5	94.2	5.8
Sodium (mg)	1446.01 (487.95)	1176.42 (187.03)	0.28	42.4	87.7	12.3
Retinol (UI)	496.30 (309.87)	390.68 (99.12)	0.52	46.3	89.0	11.0
Vitamin A (UI)	740.33 (245.29)	838.27 (160.41)	0.41	43.6	86.5	13.6
Vitamin B1 (mg)	1.41 (0.22)	1.35 (0.20)	0.33	38.7	80.9	19.1
Vitamin B3 (mg)	18.98 (5.41)	17.59 (2.49)	0.58	56.1	90.6	9.4
Vitamin B6 (mg)	2.15 (0.42)	2.08 (0.37)	0.65	49.6	87.9	12.1
Vitamin B12 (mg)	5.89 (2.62)	4.73 (1.02)	0.33	42.6	86.0	14.0
Vitamin D (mcg)	4.17 (1.85)	3.42 (1.15)	0.59	54.3	89.1	10.9
Vitamin E (mg)	7.02 (1.84)	5.89 (0.99)	0.29	43.9	87.1	12.9
Added sugar (g)	33.25 (25.28)	47.48 (29.60)	0.26	36.1	82.8	17.3
Food groups (g)						
Dairy	258.41 (205.45)	142.27 (119.60)	0.42	52.0	90.7	9.3
Meat and fishes	260.44 (110.37)	171.08 (79.82)	0.36	44.7	90.0	10.0
Cereals and legumes	310.24 (115.70)	262.29 (96.73)	0.38	41.3	81.3	18.7
Fats and oils	16.68 (11.23)	6.85 (7.48)	0.34	50.0	90.0	10.0
Fruits	456.77 (275.19)	204.42 (158.08)	0.62	49.3	92.0	8.0
Vegetables	224.21 (117.80)	139.13 (93.92)	0.42	46.7	88.0	12.0
Beverages^†^	530.68 (423.78)	442.07 (204.88)	0.41	46.0	90.0	10.0
Other food	121.97 (60.01)	79.73 (61.01)	0.35	40.0	85.3	14.7
Food processing (g)						
*In natura/minimally*	2721.04 (618.46)	1019.47 (201.09)	0.36	43.3	88.6	11.4
Culinary ingredients	34.19 (31.75)	12.50 (12.65)	0.36	34.7	84.7	14.3
Processed	187.41 (217.43)	130.13 (169.36)	0.54	46.0	82.0	18.0
Ultra-processed	340.23 (235.23)	289.79 (163.70)	0.60	46.7	92.0	8.0
**Mean**	**-**	**-**	**0.44**	**45.5**	**88.1**	**11.9**

*CUME Project, 2019*.

* *Values adjusted according to the energy*;

** *Values disattenuated and adjusted by energy values; Without including water was not measured in the 24hRs; All correlations present statistical significance (p < 0.05)*.

**Figure 2:**
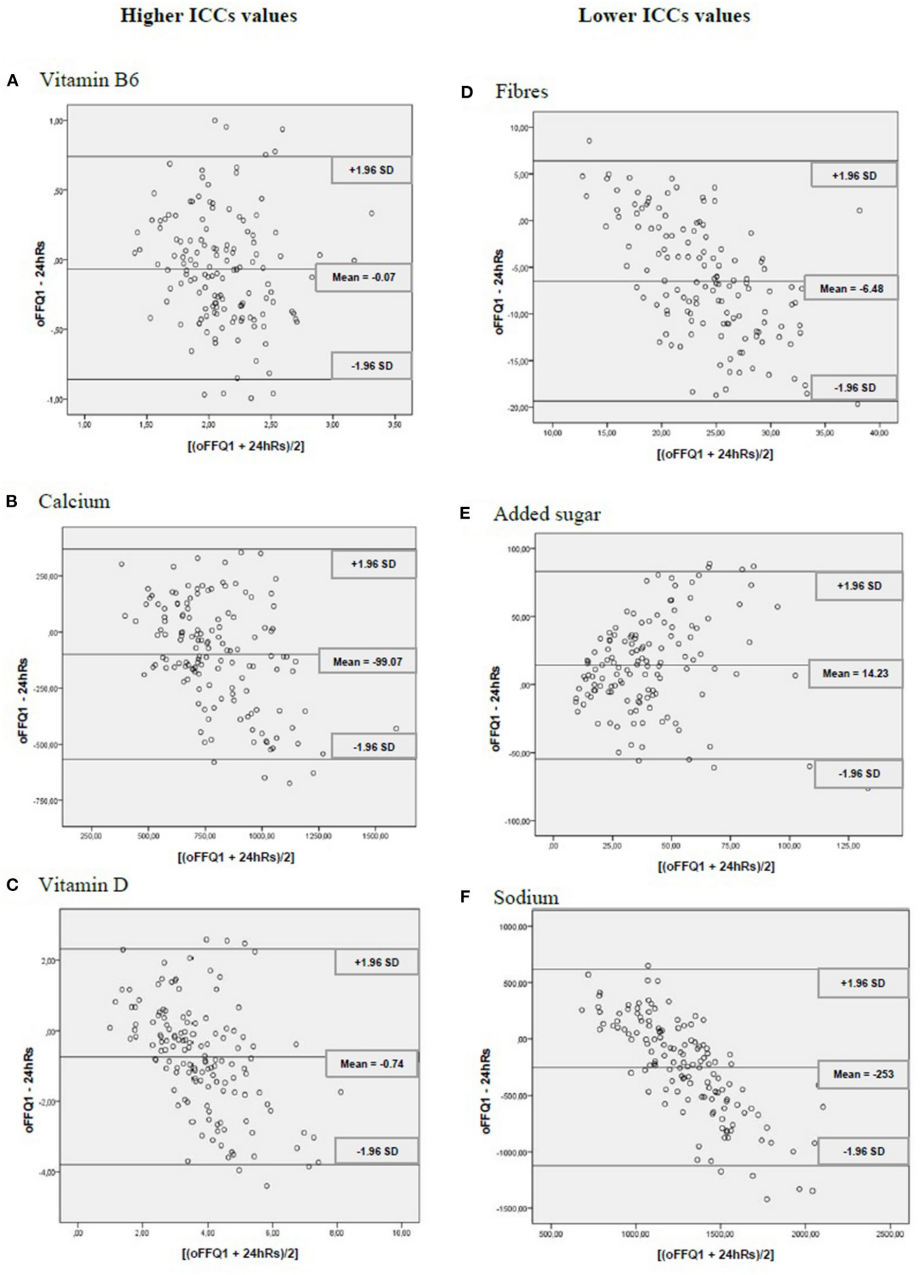
Dispersion analyses for vitamin B6, calcium, vitamin D, fiber, added sugar, and sodium between the online food frequency questionnaire (oFFQ1) and the 24-h recall (24 h Rs) (*n* = 146 participants). CUME Project, 2019. ICCs, intraclass intervals. SD, standard deviation.

Moreover, the concordance analysis of Bland and Altman showed that data were homogeneous (Figure 2).

Regarding reproducibility, the means of consumption of energy, nutrients, and food groups were similar. Overall, an agreement between the oFFQ1 and the oFFQ2 was excellent, with an average ICC of 0.76 and the exact + adjacent percentage agreement of 92.5%. The energy and all evaluated items showed moderate-to-excellent agreement, with ICCs ranging from 0.60 (vegetables) to 0.91 (vitamin E and retinol) (Table 3).

**Table 3:** Mean and SD of daily consumption, intraclass correlation coefficient (ICC), and percentage (%) of concordance between the online food frequency questionnaire 1 (oFFQ1) and the online food questionnaire 2 (oFFQ2) (*n* = 108 participants).

**Energy and nutrients(*n* = 108)**	**Mean (SD)**		**ICC**	**Concordance (%)**		
	oFFQ1^*^	oFFQ2^*^	**Adjusted values**	**Exact**	**Exact + adjacent**	**Discordant**
Energy (kcal)	2399.74 (899.13)	2274.25 (856.16)	0.80	49.0	91.0	9.0
Lipids (g)	95.71 (18.67)	91.65 (17.30)	0.64	50.0	89.7	10.4
Proteins (g)	98.14(21.44)	90.24 (19.54)	0.78	53.1	94.8	5.2
Carbohydrates (g)	256.44 (55.64)	240.22 (52.53)	0.66	60.0	90.4	9.6
Fibers (g)	28.73 (8.79)	26.21 (8.55)	0.85	70.3	96.1	4.0
Calcium (mg)	852.38 (275.26)	782.13 (252.17)	0.73	54.8	93.3	6.7
Phosphorus (mg)	1446.67 (275.55)	1332.67 (270.22)	0.75	55.6	91.9	8.1
Iron (mg)	12.39 (2.23)	11.42 (2.15)	0.68	53.1	91.8	8.2
Sodium (mg)	1403.82 (491.41)	1314.64 (557.49)	0.67	50.5	92.9	7.1
Retinol (UI)	444.73 (231.90)	435.31 (231.15)	0.91	50.5	96.7	3.3
Vitamin A (UI)	762.82 (295.97)	753.69 (288.24)	0.86	55.7	94.9	5.2
Vitamin B1 (mg)	1.38 (0.18)	1.30 (0.20)	0.62	42.3	89.7	10.4
Vitamin B3 (mg)	18.35 (4.97)	17.69 (5.44)	0.80	60.0	93.0	7.0
Vitamin B6 (mg)	2.11 (0.41)	1.97 (0.38)	0.71	52.0	93.2	6.8
Vitamin B12 (mg)	5.95 (2.99)	5.54 (3.06)	0.90	66.7	96.8	4.2
Folate (mcg)	379.76 (102.15)	351.26 (101.85)	0.78	63.1	93.2	6.8
Vitamin D (mcg)	4.05 (3.69)	3.69 (2.03)	0.88	56.7	98.9	2.1
Vitamin E (mg)	7.28 (2.39)	7.29 (2.3)	0.91	61.5	96.9	3.1
AG Trans (g)	3.23 (1.59)	3.00 (1.52)	0.82	56.3	93.8	6.2
Added sugar (g)	29.99 (18.73)	23.10 (15.30)	0.77	63.9	90.7	9.3
Food groups (g)						
Dairy	252.27 (211.53)	216.14 (213.41)	0.62	57.4	92.6	7.4
Meat and fishes	250.93 (117.41)	227.22 (100.45)	0.69	41.7	89.9	10.1
Cereals and legumes	321.66 (114.04)	201.97 (87.63)	0.74	48.2	88.9	11.1
Fats and oils	17.28 (11.30)	16.58 (12.88)	0.83	52.8	93.6	5.4
Fruits	440.05 (280.22)	424.66 (241.01)	0.83	57.4	90.7	9.3
Vegetables	213.52 (112.64)	287.62 (127.63)	0.60	49.1	89.9	10.1
Beverages	1604.89 (531.06)	1645.04 (575.45)	0.75	57.4	94.5	5.5
Other food	115.79 (69.30)	95.06 (49.74)	0.57	44.4	81.5	18.5
Food processing (g)						
*In natura/minimally*	2738.50 (711.03)	2679.26 (756.02)	0.81	54.6	91.6	8.4
Culinary ingredients	29.95 (19.77)	27.87 (19.46)	0.75	54.6	93.5	6.5
Processed	148.71 (113.11)	141.47 (103.88)	0.82	55.6	94.4	5.6
Ultra-processed	325.99 (223.40)	277.22 (178.22)	0.82	46.3	90.7	9.3
**Mean**	-	-	**0.76**	**54.5**	**92.5**	**7.5**

*CUME Project, 2019*.

* *Values adjusted by energy; All correlations presented statistical significance (p < 0.05)*.

## Discussion

In this study, we demonstrated that the oFFQ used at the CUME had moderate validity and excellent reproducibility for total energy consumption and most nutrients and food groups.

Regarding validity, compared with the 24hRs, the oFFQ presented moderate ICC values for most of the items evaluated, results that were similar to the previous national (2, 29) and international (30, 31) studies.

These ICC values may be influenced by the fact that most of the means of energy, nutrients, and food group consumption in the oFFQ1 were higher than those measured in the 24hRs. In this sense, 24hRs provide more detailed and less biased data than FFQ (32). In general, the FFQ overestimates food consumption (33–35).

Although the ICCs of some micronutrients and food groups were low for some of them (iron, vitamin B1, vitamin B12, meat and fish, cereals and legumes, fats and oils, other foods, fresh/minimally processed foods, and culinary ingredients), the values were very close to the limit considered as moderate (0.40); furthermore, the average of the exact + adjacent tertiles agreement was high (88.1%) with all items showing a percentage higher than 80%, a result also close to those observed in Brazilian studies on the subject (2, 36). Interestingly, the mean differences by the Bland and Altman method were minor, and the data were homogeneous. These findings were consistent with those evidenced in a cohort study conducted with a sample of Brazilian middle-aged adults (2).

Low ICCs of some micronutrients and food groups may have been influenced by the following factors: (a) the FFQ consists of a set list of food items. At the same time, the 24hRs allow quantifying all foods and beverages consumed in the period before the interview. This potentially causes consumption overestimation in the FFQ due to the tendency of the participants to indicate greater food intake and rare items in their daily diet (37). The higher averages of energy consumption can reinforce this statement. Most of the nutrients and food groups were measured by the oFFQ in relation to the 24hRs; (b) we used the Multiple Pass method (20) to apply the 24hRs and minimize errors in measuring the diet. This method allowed greater detail of the foods consumed and investigation of sugar or sweetener addition to coffee, tea, milk, and juice, as well as the use of spices, sauces, and olive oil in vegetables, which may have reflected in higher consumption averages of these items in the 24hRs; (c) a higher average consumption of fiber in the oFFQ compared with the 24hRs could be explained by the intake overestimation of source foods considered socially approved, such as fruits, vegetables, and legumes (38); and (d) 24hRs is a method often used to validate the FFQ; however, it presents memory bias and errors when estimating the portion size.

In addition to the validity and reproducibility of nutrients, the present study extrapolated the assessment to food groups since individuals do not consume only isolated nutrients but meals consisting of food and nutrients (39). Therefore, validity and reproducibility by food groups have been carried out, considering only nutritional similarity as a grouping criterion (40). Still, to our knowledge, this is the first study assessing the validity and reproducibility of food groups according to the degree of industrial processing (41).

The oFFQ used at CUME proved to be valid compared with the 24hRs, showing that such a questionnaire can measure what it is intended to measure (29, 42). This result is important not only for this study, but it also has an impact on the nutritional epidemiology and public health areas, since instruments for data collection in the virtual environment (Internet) are very useful and practical in countries with a large geographical extension, such as Brazil, dismissing face-to-face meetings between researchers and participants (6, 7).

Regarding reproducibility, when compared with the oFFQ2, the oFFQ1 presented excellent ICC values in most of the items evaluated, and these results are congruent with those observed in studies on the same subject (29, 42); furthermore, in the analysis based on tertiles, the average of the exact + adjacent agreement of the comparison between the oFFQ1 and the oFFQ2 was almost perfect (92.5%), being this result similar to those evidenced in other studies (2, 37).

Reproducibility is the ability of an instrument to produce similar estimates in two different moments with the same accuracy (29); therefore, the fact that the oFFQ showed to be reproducible is fundamental for the CUME project because evaluations of the diet and food intake of the participants will be carried out on different occasions over time due to its longitudinal design.

To ensure the quality of the information, some precautions were necessary for carrying out the study, such as assessment of seasonal food consumption, with data collection of the 24hRs carried out in two different seasons of the year; applying the second oFFQ in a timely manner to avoid fundamental changes in diet or remembering the answers given in the first questionnaire (43); prior training for all nutritionists with a standardized script using the Multiple-Pass method (20); sending a photo album with utensils and homemade measures to aid participants during the food survey (36). Additionally, it is an innovative study because the validity and reproducibility of food have been carried out according to industrial processing.

Nonetheless, this study also has limitations. We performed data collection of the 24hRs in two seasons of the year (winter and autumn), not considering dietary variability throughout all year. However, other validation studies also opted for the application of two 24hRs with shorter intervals (43, 44) to improve study adherence. The 24hRs interview, including two non-consecutive days, is recommended in the European Food Safety Authority guidance on the European Union menu methodology for nationwide individual food consumption studies (45) and endorsed by researchers of the European Food Consumption Survey Methods group (46). Besides, this study innovated by encompassing the collection of four 24hRs in two different seasons; 12 participants (8.2%) did not respond to the two 24hRs applied in the second moment of the validation study, which could be compromising the assessment of food consumption variability of these participants; there was a loss of participants between the stages of validity and reproducibility of the oFFQ, which could reduce the power of the statistical tests. On the other hand, this fact does not seem to have occurred since all the ICCs were significant and with higher values in the reproducibility stage.

Finally, it is worth highlighting some advantages of the oFFQ method, such as low cost, simple analysis, easy application, does not modify consumption over time and can classify individuals according to their usual eating patterns and to associate them to health conditions, which makes it feasible for their use in population studies (2, 47).

## Conclusions

When evaluating the results presented, it is concluded that the FFQ developed by the CUME that could be completed online can be used with satisfactory validity and reproducibility to analyze the association between food consumption and NCDs in adults with a high level of education, the target population of the CUME project; however, correction factors must be applied to some nutrients and food groups in future data analysis of the project involving food consumption.

## Data Availability Statement

The datasets presented in this article are not readily available because, Data belongs to a still ongoing longitudinal project involving several academic institutions, and therefore cannot be shared just yet. Requests to access the datasets should be directed to Adriano Marçal Pimenta, adrianomp@ufmg.br.

## Ethics Statement

The studies involving human participants were reviewed and approved by protocol n° 596.741-0/2013. The patients/participants provided their written informed consent to participate in this study.

## Author Contributions

AP, HH, and JB conceived the study, analyzed the data, and drafted and revised the final version of the manuscript. HA, JM-R, AM, and LS analyzed the data and drafted and revised the final version of the manuscript. AGD, AL, and FO drafted and revised the final version of the manuscript. All authors contributed to the article and approved the submitted version.

## Funding

This research was funded by the FAPEMIG (State of Minas Gerais, Brazil), grant numbers (CDS-APQ00571/13, CDS-APQ-02407/16, CDS-APQ-00424/17). AP, HH, FO, and JB are research productivity fellows of the CNPq (Ministry of Science and Technology, Brazil). JM-R has received a postdoctoral fellowship from the CAPES (Ministry of Science and Technology, Brazil). AM and AGD has received postdoctoral fellowships from the CAPES. HA has received master's fellowship from the CAPES, and AL is a recipient of PhD fellowship from the CAPES.

## Conflict of Interest

The authors declare that the research was conducted in the absence of any commercial or financial relationships that could be construed as a potential conflict of interest.

## Publisher's Note

All claims expressed in this article are solely those of the authors and do not necessarily represent those of their affiliated organizations, or those of the publisher, the editors and the reviewers. Any product that may be evaluated in this article, or claim that may be made by its manufacturer, is not guaranteed or endorsed by the publisher.

## References

[1] Schneider BC Motta JVDS Muniz LC Bielemann RM Madruga SW Orlandi SP. Desenho de um questionário de frequência alimentar digital autoaplicado para avaliar o consumo alimentar de adolescentes e adultos jovens: Coortes de nascimentos de Pelotas, Rio Grande do Sul. Rev Bras Epidemiol. (2016) 19:419–32. 10.1590/1980-5497201600020017 27532763

[2] Molina M del CB Benseñor IM Cardoso L de O Velasquez-Melendez G Drehmer M Pereira TSS. Reprodutibilidade e validade relativa do Questionário de Frequência Alimentar do ELSA-Brasil. Cad Saude Publica. (2013) 29:379–89. 10.1590/S0102-311X2013000600024 23459823

[3] Galea S Tracy M. Participation rates in epidemiologic studies. Ann Epidemiol. (2007) 17:643–53. 10.1016/j.annepidem.2007.03.013 17553702

[4] Van Gelder MMHJ Bretveld RW Roeleveld N. Web-based questionnaires: the future in epidemiology? Am J Epidemiol. (2010) 172:1292–8. 10.1093/aje/kwq291 20880962

[5] Illner AK Freisling H Boeing H Huybrechts I Crispim SP Slimani N. Review and evaluation of innovative technologies for measuring diet in nutritional epidemiology. Int J Epidemiol. (2012) 41:1187–203. 10.1093/ije/dys105 22933652

[6] García-Segovia P González-Carrascosa R Martínez-Monzó J Ngo J Serra-Majem L. Nuevas tecnologías aplicadas a los cuestionarios de frecuencia de consumo de alimentos: Una perspectiva actual. Nutr Hosp. (2011) 26:803–6. 10.3305/nh.2011.26.4.5154 22470027

[7] Vergnaud AC Touvier M Méjean C Kesse-Guyot E Pollet C Malon A. Agreement between web-based and paper versions of a socio-demographic questionnaire in the NutriNet-Santé study. Int J Public Health. (2011) 56:407–17. 10.1007/s00038-011-0257-5 21538094

[8] Szwarcwald CL Souza Júnior PRB Damacena GN Malta DC Barros MBA Romero DE. ConVid—behavior survey by the internet during the COVID-19 pandemic in Brazil: conception and application methodology. Cad Saude Publica. (2021) 37:e00268320. 10.1590/0102-311X00268320 33950078

[9] Francisco PMSB Assumpção D Malta DC. Co-occurrence of smoking and unhealthy diet in the Brazilian adult population. Arq Bras Cardiol. (2019) 113:699–709. 10.5935/abc.20190222 31691752 PMC7020877

[10] Gomes Domingos AL Miranda AE da S Pimenta AM Hermsdorff HHM Oliveira FLP de dos Santos LC. Cohort profile: the cohort of universities of Minas Gerais (CUME). Int J Epidemiol. (2018) 47:1743–4. 10.1093/ije/dyy152 30060144

[11] Slater B Philippi ST Marchioni DML Fisberg RM. Validation of food frequency questionnaires—FFQ: methodological considerations. Rev Bras Epidemiol. (2003) 6:200–8.

[12] Hercberg S. Web-based studies: the future in nutritional epidemiology (and overarching epidemiology) for the benefit of public health? Prev Med. (2012) 55:544–5. 10.1016/j.ypmed.2012.09.016 23010436

[13] Fisberg RM Marchioni DML Colucci ACA. Avaliação do consumo alimentar e da ingestão de nutrientes na prática clínica. Arq Bras Endocrinol Metabol. (2009) 53:617–24. 10.1590/s0004-27302009000500014 19768252

[14] Seguí-Gómez M de la Fuente C Vázquez Z de Irala J Martínez-González MA. Cohort profile: the ‘Seguimiento Universidad de Navarra' (SUN) study. Int J Epidemiol. (2006) 35:1417–22. 10.1093/ije/dyl223 17060332

[15] Henn RL Fuchs SC Fuchs FD. Development and validation of a food frequency questionnaire (FFQ-Porto Alegre) for adolescent, adult and elderly populations from Southern Brazil. Cad Saude Publica. (2010) 26:2068–79. 10.1590/s0102-311x2010001100008 21180980

[16] Coelho AB de Aguiar DRD Fernandes EA. Padrão de consumo de alimentos no Brasil. Rev Econ e Sociol Rural. (2009) 47:335–62. 10.1590/s0103-20032009000200002

[17] Volp ACP Lacerda EMA Benzecry E. Tabela para avaliação de consumo alimentar em medidas caseiras. 5a ed. São Paulo: Atheneu São Paulo (2004).

[18] Lopez RPS Botelho RBA. Álbum fotográfico de porções alimentares. 1a ed. São Paulo: Metha (2013).

[19] Miyamura PC. AR Desenvolvimento de registro fotográfico de alimentos e preparações referidos por pacientes em acompanhamento nutricional. Nutr Bras. (2015) 14:85–9.

[20] Conway JM Ingwersen LA Vinyard BT Moshfegh AJ. Effectiveness of the US department of agriculture 5-step multiple-pass method in assessing food intake in obese and non-obese women. Am J Clin Nutr. (2003) 77:1171–8. 10.1093/ajcn/77.5.1171 12716668

[21] Siqueira JH Mill JG Velasquez-Melendez G Moreira AD Barreto SM Benseñor IM. Sugar-sweetened soft drinks and fructose consumption are associated with hyperuricemia: cross-sectional analysis from the Brazilian longitudinal study of adult health (ELSA-Brasil). Nutrients. (2018) 10:1–15. 10.3390/nu10080981 30060512 PMC6116015

[22] Instituto Brasileiro de Geografia e Estatística. Demographic Census. (2010). Available online at: https://censo2010.ibge.gov.br/.

[23] Monteiro CA Cannon G Moubarac JC Levy RB Louzada MLC Jaime PC. The UN decade of nutrition, the NOVA food classification and the trouble with ultra-processing. Public Health Nutr. (2018) 21:5–17. 10.1017/S1368980017000234 28322183 PMC10261019

[24] Nusser SM Carriquiry AL Dodd KW Fuller WA. A semiparametric transformation approach to estimating usual daily intake distributions. J Am Stat Assoc. (2016) 91:1440–9. 10.1080/01621459.1996.10476712

[25] Nusser SM Fuller WA Guenther PM. Estimating Usual Dietary Intake Distributions: Adjusting for Measurement Error and Non-normality in 24-Hour Food Intake Data. Staff Report 95-SR 80 (1995).

[26] Willett WC Stampfer M. Total energy intake: implications for epidemiologic analysis. Am J Epidemiol. (1986) 1:17–27. 10.1093/oxfordjournals.aje.a114366 3521261

[27] Kramer M Feinstein A. Biostatistics of concordance. Clin Pharmacol Ther. (1981) 29:111–23. 10.1038/clpt.1981.18 7460469

[28] Bland J Altman DG. Statistical methods for assessing agreement between two methods of clinical measurement. Lancet. (1986) 1:307–10. 10.1016/S0140-6736(86)90837-8 2868172

[29] Bonatto S Henn RL Olinto MTA dos Anjos LA Wahrlich V Waissmann W. Reproducibility, relative validity, and calibration of a food-frequency questionnaire for adults in Greater Metropolitan Porto Alegre, Rio Grande do Sul State, Brazil. Cad Saude Publica. (2014) 30:1837–48. 10.1590/0102-311X00151313 25317513

[30] Nyström CD Henriksson H Alexandrou C Bergström A Bonn S Bälter K. Validation of an online food frequency questionnaire against doubly labelled water and 24 h dietary recalls in pre-school children. Nutrients. (2017) 9:66. 10.3390/nu9010066 28098765 PMC5295110

[31] Rodriguez CA Smith ER Villamor E Zavaleta N Respicio-Torres G Contreras C. Development and validation of a food frequency questionnaire to estimate intake among children and adolescents in Urban Peru. Nutrients. (2017) 9:1–10. 10.3390/nu9101121 29036893 PMC5691737

[32] Park Y Dodd KW Kipnis V Thompson FE Potischman N Schoeller DA. Comparison of self-reported dietary intakes from the automated self-administered 24-h recall, 4-d food records, and food-frequency questionnaires against recovery biomarkers. Am J Clin Nutr. (2018) 107:80–93. 10.1093/ajcn/nqx002 29381789 PMC5972568

[33] Steinemann N Grize L Ziesemer K Kauf P Probst-Hensch N Brombach C. Relative validation of a food frequency questionnaire to estimate food intake in an adult population. Food Nutr Res. (2017) 61:1305193. 10.1080/16546628.2017.1305193 28469546 PMC5404419

[34] Yuan C Spiegelman D Rimm EB Rosner BA Stampfer MJ Barnett JB. Validity of a dietary questionnaire assessed by comparison with multiple weighed dietary records or 24-hour recalls. Am J Epidemiol. (2017) 185:570–84. 10.1093/aje/kww104 28338828 PMC5859994

[35] Mumu SJ Merom D Ali L Fahey PP Hossain I Rahman AKMF. Validation of a food frequency questionnaire as a tool for assessing dietary intake in cardiovascular disease research and surveillance in Bangladesh. Nutr J. (2020) 19:42. 10.1186/s12937-020-00563-7 32410632 PMC7227307

[36] Brito AP Araujo MC Guimarães CP Pereira RA. Relative validity of a food frequency questionnaire supported by images. Cienc e Saude Coletiva. (2017) 22:457–68. 10.1590/1413-81232017222.26392015

[37] Selem SS de C de Carvalho AM Verly-Junior E Carlos JV Teixeira JA Marchioni DML. Validade e reprodutibilidade de um questionário de frequência alimentar para adultos de São Paulo, Brasil. Rev Bras Epidemiol. (2014) 17:852–9. 10.1590/1809-4503201400040005 25388486

[38] Hu FB. Dietary pattern analysis: a new direction in nutritional epidemiology. Curr Opin Lipidol. (2002) 13:3–9. 10.1097/00041433-200202000-00002 11790957

[39] Machado FC de S Henn RL Olinto MTA Anjos LA Wahrlich V Waissmann W. Reprodutibilidade e validade de um questionário de freqüência alimentar baseado em grupos de alimentos, em população adulta da região metropolitana de porto Alegre, RS. Rev Nutr. (2012) 25:65–77. 10.1590/S1415-52732012000100007

[40] Brasil. Ministério da Saúde. Secretaria de Vigilância em Saúde. Guia Alimentar para a População Brasileira Guia Alimentar para a População Brasileira. Brazil: Ministério da Saúde (2014).

[41] Marques RDMB Oliveira AC De Teles SADS Stringuini MLF Fornés NS Gardenghi G. Relative validity and reproducibility of a quantitative food frequency questionnaire for adolescents with type 1 diabetes: validity of a food frequency questionnaire. Int J Endocrinol. (2014) 2014:976508. 10.1155/2014/976508 25250051 PMC4163310

[42] Cade J Thompson R Burley V Warm D. Development, validation and utilisation of food-frequency questionnaires—a review. Public Health Nutr. (2002) 5:567–87. 10.1079/phn2001318 12186666

[43] Denova-Gutiérrez E Ramírez-Silva I Rodríguez-Ramírez S Jiménez-Aguilar A Shamah-Levy T Rivera-Dommarco JA. Validity of a food frequency questionnaire to assess food intake in Mexican adolescent and adult population. Salud Publica Mex. (2016) 58:617–28. 10.21149/spm.v58i6.7862 28225938

[44] Bijani A Esmaili H Ghadimi R Babazadeh A Rezaei RG Cumming R. Development and validation of a semi-quantitative food frequency questionnaire among older people in north of Iran. Caspian J Intern Med. (2018) 9:78–86. 10.22088/cjim.9.1.78 29387324 PMC5771365

[45] European Food Safety Authority 2014. Guidance on the EU menu methodology. EFSA J. (2014) 12:3944. 10.2903/j.efsa.2014.3944

[46] Brussaard JH Johansson L Kearney J EFCOSUM Group. Rationale and methods of the EFCOSUM project. Eur J Clin Nutr. (2002) 56:S4–7. 10.1038/sj.ejcn.1601422 12082511

[47] Santana JDM Camilo VMA DE Freitas FVN Da Silva IDMM Da Silva DF De Oliveira FS. Desenvolvimento De Questionário De Frequência Alimentar Para População Adulta Residentes Em Santo Amaro, Bahia, Brasil. DEMETRA Aliment Nutr Saúde. (2016) 11:195–210. 10.12957/demetra.2016.18460

