# Supplementary material for: Online Food Frequency Questionnaire From the Cohort of Universities of Minas Gerais (CUME Project, Brazil): Construction, Validity, and Reproducibility
Source: Front Nutr. 2021 Sep 23;8:709915. doi: 10.3389/fnut.2021.709915 (PMC8495245; doi:10.3389/fnut.2021.709915)
Supplement: Supplementary file 1 [file Table_1.DOCX]

**Table S1** Food groups according to the degree of processing^*^. CUME Project, 2019

| **Food groups** | **Food items** |
| --- | --- |
| ***In natura/minimally processed*** | |
| Rice and pastas | White rice, brown rice, pasta, gnocchi, soup (rice/pasta) |
| Legumes | Beans/lentils, chickpeas |
| Cereals, roots and tubers | Green corn, *canjiquinha*, cooked corn meal (*polenta*/*angu*), cassava/yam/*baroa* potato, cassava flour/breadcrumbs, corn flour, cooked potato, oat/wheat germ/granola |
| Fried food | French fries, fried cassava, fried *polenta* |
| Meat and entrails | Beef steak, diced beef, pork, meatballs, chicken with skin, chicken without skin, salmon, liver/gizzard/heart, mutton, soy meat, salmon, other fish, shrimp/seafood, sushi/sashimi |
| Eggs | Cooked chicken egg |
| Fresh dairy | Whole milk, skimmed milk, semi-skimmed milk |
| Fruits | Banana, orange/tangerine, apple/pear, avocado, pineapple, açai (pulp), acerola, guava, kiwi, papaya, mango, watermelon, melon, strawberry/cherry, peach/plum/nectarine, grape, raisin, tropical fruits (*pitanga,* mangosteen, soursop, *umbu, cupuaçu*), fruit salad |
| Vegetables | Lettuce/chard, watercress/kale/arugula/spinach/chicory, pumpkin/squash, zucchini/chayote, eggplant, beetroots, carrots, cauliflower/cabbage, cucumber, red/green peppers, tomatoes, green beans, vegetable soup, pepper |
| Olive oil, nuts and seeds | Peanuts/walnuts/Brazil nuts/cashew nuts |
| Fresh beverages | Coffee, mate tea/black tea, white/green tea, natural juice, *mate* |
|  |  |
| **Culinary ingredients** | Olive oil, soy oil, sunflower oil, butter, sugar, brown sugar, sweetener, honey, salt, light sugar, canola oil, pork fat |
|  | |
| **Processed** | |
| Processed cheeses | Cheese (mozzarella/provolone/*minas* cheese, *canastra* cheese, *prato* cheese, ricotta, cottage |
| Processed meats | Biltong, smoked meat, sardines, cod |
| French bread | French bread |
| Processed sweet food | Fruit jelly, guava/peach/fig/marmalade, syrup fruits |
| Fermented alcoholic beverages | Beer, wine, other wines |
|  | |
| **Ultra-processed** | |
| Ultra-processed dairy | Curd, light curd, whole yogurt, low-fat/light yogurt |
| Embedded foods | Mortadella/salami/ham/turkey breast/turkey, sausage/grilled sausage/lard |
| Ultra-processed bread | Loaf of bread, toast, cheese bread, sweet bread, brown bread (rye/wheat/oats), light bread, breakfast cereal |
| Margarine | Margarine, light mayonnaise, mayonnaise |
| Sweeten beverages | Soda, diet/zero soda, industrialized juice (canned/box/powder), diet and/or light industrialized juice |
| Distilled alcoholic beverages | vodka/rum/whisky, *cachaça* (sugar cane spirit) |
| Fast food and ultra-processed sweets | Pizza, hot dog/beef burger/chicken fried snacks (*coxinha/pastel*/*rissole*/croquette), slapstick/pie/quiche, popcorn, industrialized chips, lasagne/cannelloni/rondelle, ice cream, light ice cream, soy milk, dark /*brigadeiro* (Brazilian chocolate truffle), cereal bar, chocolate pudding/ambrosia/dulce de leche/rice pudding/flan, sweets/*maria mole (Brazilian treat that resembles a marshmallow)*/candy, chocolate (50-70% cocoa), milk chocolate/sugarplum mustard. |

* Monteiro CA, Cannon G, Moubarac JC, Levy RB, Louzada MLC, Jaime PC. The UN Decade of Nutrition, the NOVA food classification and the trouble with ultra-processing. Public Health Nutr. 2018; 21(1): 5-17.
